# Further evidence that mechanisms of host/symbiont integration are dissimilar in the maternal versus embryonic *Acyrthosiphon pisum* bacteriome

**DOI:** 10.1186/s13227-020-00168-5

**Published:** 2020-11-10

**Authors:** Celeste R. Banfill, Alex C. C. Wilson, Hsiao-ling Lu

**Affiliations:** 1 Department of Biology, University of Miami, Coral Gables, FL 33146 USA; 2 Department of Biotechnology, National Formosa University, Huwei, Taiwan

**Keywords:** Host/symbiont developmental integration, Endosymbiosis, Bacteriome, Amino acid transporter, Coevolution

## Abstract

**Background:**

Host/symbiont integration is a signature of evolutionarily ancient, obligate endosymbioses. However, little is known about the cellular and developmental mechanisms of host/symbiont integration at the molecular level. Many insects possess obligate bacterial endosymbionts that provide essential nutrients. To advance understanding of the developmental and metabolic integration of hosts and endosymbionts, we track the localization of a non-essential amino acid transporter, ApNEAAT1, across asexual embryogenesis in the aphid, *Acyrthosiphon pisum*. Previous work in adult bacteriomes revealed that ApNEAAT1 functions to exchange non-essential amino acids at the *A. pisum*/*Buchnera aphidicola* symbiotic interface. Driven by amino acid concentration gradients, ApNEAAT1 moves proline, serine, and alanine from *A. pisum* to *Buchnera* and cysteine from *Buchnera* to *A. pisum*. Here, we test the hypothesis that ApNEAAT1 is localized to the symbiotic interface during asexual embryogenesis.

**Results:**

During *A. pisum* asexual embryogenesis, ApNEAAT1 does not localize to the symbiotic interface. We observed ApNEAAT1 localization to the maternal follicular epithelium, the germline, and, in late-stage embryos, to anterior neural structures and insect immune cells (hemocytes). We predict that ApNEAAT1 provisions non-essential amino acids to developing oocytes and embryos, as well as to the brain and related neural structures. Additionally, ApNEAAT1 may perform roles related to host immunity.

**Conclusions:**

Our work provides further evidence that the embryonic and adult bacteriomes of asexual *A. pisum* are not equivalent. Future research is needed to elucidate the developmental time point at which the bacteriome reaches maturity.

## Background

Understanding the cellular and developmental integration of hosts and symbionts is important for understanding how endosymbioses function and are maintained [1–7]. Many insects feed on nutritionally challenging diets, such as plant sap or blood. Plant sap is low in essential amino acids and blood is low in B vitamins; each of these deficiencies limits insect growth and/or reproduction [8–11]. The acquisition of endosymbiotic bacteria that provision hosts with essential amino acids and/or vitamins ensures the persistence of insects on growth-limiting diets [12, 13]. Here, we use the model nutritional symbiosis between aphids and their bacterial endosymbiont, *Buchnera aphidicola*, to elucidate cellular mechanisms underlying host/symbiont integration across embryonic development.

The aphid/*Buchnera* symbiosis is ancient [14]. Over evolutionary time, aphids and *Buchnera* have become developmentally and metabolically integrated to such an extent that aphids cannot reproduce without *Buchnera*, and *Buchnera* cannot live outside their host [1]. Like all vertically transmitted endosymbionts, the fitness of *Buchnera* is tightly coupled to that of its host [15, 16]. *Buchnera* reside in specialized host cells called bacteriocytes, that aggregate to form an organ called the bacteriome [12, 17]. Studying the developmental integration of *Buchnera* involves determining the mechanisms of transmission, bacteriocyte cellularization and bacteriome maturation, as well as how the function of the symbiosis changes across host development. As intracellular symbionts, *Buchnera* critically depend on their host bacteriocyte cells for energy and metabolic precursors that include most but not all non-essential amino acids [1, 18, 19]. In return, *Buchnera* compensate for dietary shortfalls in cellular and metabolic building blocks that include essential amino acids and some B vitamins [1, 11, 18]. The genomic, cellular, and molecular processes that contribute to this metabolism characterize the metabolic integration of aphids and *Buchnera*. Here, building on our earlier work, we further investigate the metabolic integration of *Buchnera* in the pea aphid, *Acyrthosiphon pisum*, across asexual embryogenesis.

Four previous studies shed light on the metabolic integration of *Buchnera* during *A. pisum* asexual embryogenesis. The first revealed that *Buchnera* proteins, including those involved in essential amino acid biosynthesis, are differentially regulated in *Buchnera* occupying the embryonic versus maternal bacteriome [20]. The second, by immunoblot analyses and immunolocalization, revealed differential localization in maternal vs. embryonic bacteriomes of RlpA4 (an *A. pisum* protein of bacterial origin) [21]. The third, which described the localization of glutamine transporter ApGLNT1 during viviparous embryogenesis, was consistent with the previous two in revealing an ontogenetic shift in bacteriome function, finding differences in transporter localization between the embryonic and maternal bacteriome [2]. In contrast, the fourth study revealed some equivalency of gene expression between the embryonic and maternal bacteriomes, showing that amino acid transporters ACYPI000536 and ACYPI008904 are important for endosymbiont integration [7], as well as maternal bacteriome function [22].

**Fig. 1:**
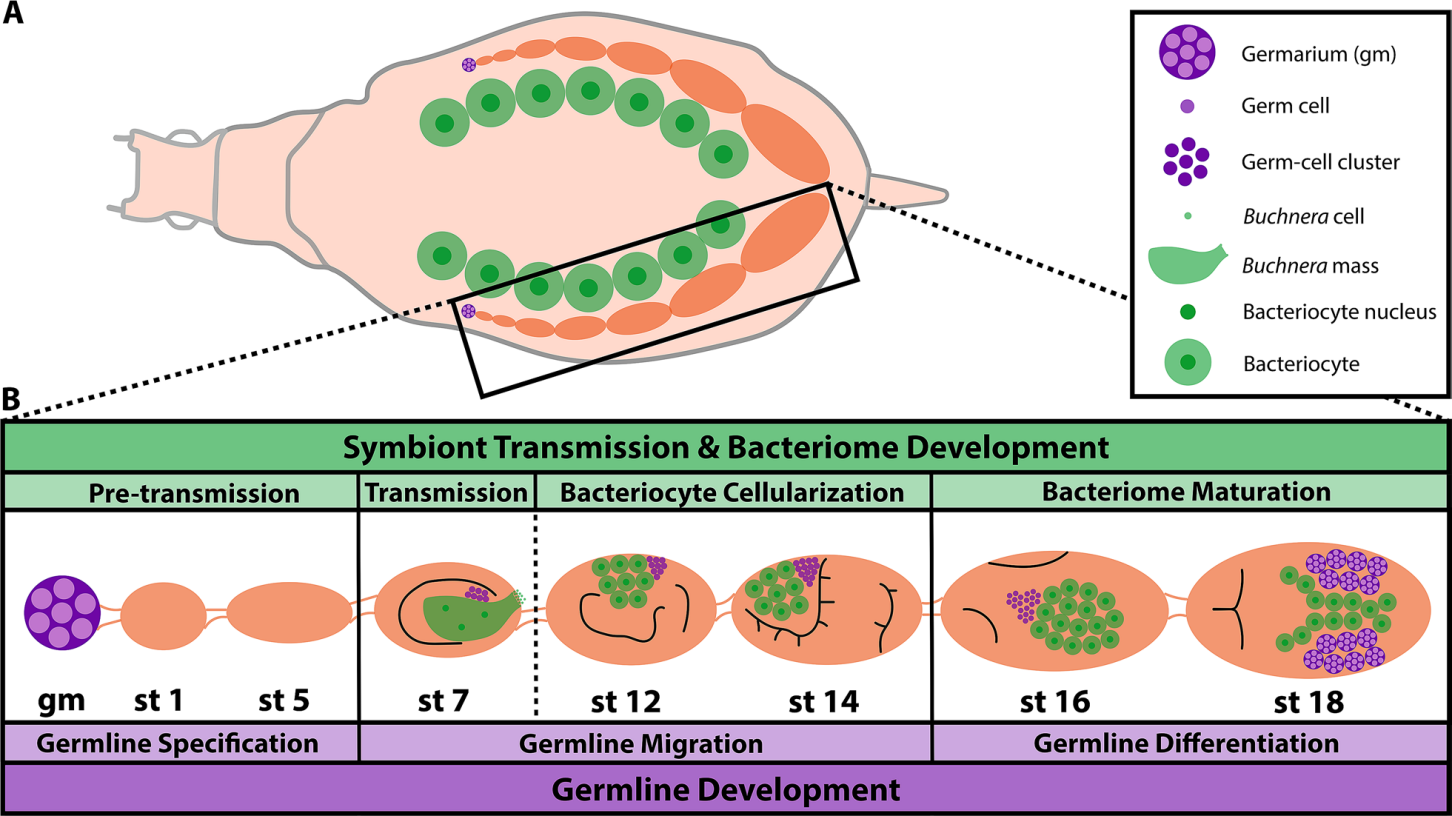
Asexual *A. pisum* embryogenesis. **a** An illustration of a dorsal view cross section of an asexual *A. pisum* adult female. The black box encloses a single ovariole (chain of embryos and oocytes). **b** A magnified view of the ovariole, containing representative stages across embryogenesis from germarium to stage 18. Symbiont transmission and development of the bacteriome (the symbiotic organ) are shown in green. Germline development is shown in purple. See the key for identification of specific structures. *gm* germarium, *st* stage. Embryo morphology in stages 7–18 is sketched in black lines (for a complete morphological description see Miura et al. [23])

As cyclical parthenogens, female aphids seasonally switch between asexual and sexual reproduction [23]. Asexual aphids are viviparous, giving birth to live young (while sexual aphids are oviparous) [24]. The integration of *Buchnera* during *A. pisum* development can be divided into four phases: pre-transmission, transmission, bacteriocyte cellularization, and bacteriome maturation [2] (see Fig. 1). All female aphids have two ovaries; in asexual *A. pisum* each ovary comprises six–seven ovarioles, with each ovariole containing a string of ~ 11 oocytes/embryos^1^ [23]. Located at the terminal end of each ovariole is the germarium. Germline development can be divided into three phases: germline specification, germline migration, and germline differentiation [23, 25–28] (see Fig. 1). Notably, two types of germaria are present within asexual females: the germaria of the F1 generation and the embryonic germaria (present within late-stage embryos) that will give rise to the F2 generation. This feature of asexual aphid embryogenesis is described as a *telescoping of generations*: viviparous aphids contain the embryos of their daughters (F1 generation) and granddaughters (F2 generation) [29].

Here, we study the localization of a recently characterized non-essential amino acid transporter, ApNEAAT1, across asexual embryogenesis in *A. pisum*. ApNEAAT1 is an exchanger of small dipolar amino acids that include serine, proline, cysteine, alanine, and glycine which in the maternal bacteriome localizes to the bacteriocyte plasma membrane and the host-derived symbiosomal membrane that separates individual *Buchnera* cells from the bacteriocyte cytoplasm [30]. Remarkably, transport by ApNEAAT1 is driven by amino acid concentration gradients and not by transmembrane ion gradients [30]. *Buchnera* has lost the ability to synthesize three amino acids transported by ApNEAAT1: proline, serine, and alanine [18, 31, 32], while transporter function implicates the supply of cysteine from *Buchnera* to *A. pisum* [33]. Given the localization of ApNEAAT1 to the bacteriocyte and symbiosomal membranes of maternal bacteriomes, *Buchnera’s* dependence on host-supplied proline, serine, and alanine, and the retention of cysteine biosynthetic capacity by *Buchnera* in the face of ongoing gene loss, it is clear that ApNEAAT1 plays an important role in maternal bacteriome function [30]. However, the role of ApNEAAT1 in the embryonic bacteriome is yet to be studied. Given that ApNEAAT1 exchanges amino acids along concentration gradients, and apparently does not serve a regulatory function, we hypothesize that ApNEAAT1 will localize to both the symbiosomal and bacteriocyte membranes of *A. pisum* asexual embryos to provision proline, serine, and alanine to *Buchnera*. Additionally, because viviparous embryos depend on maternal provisioning of nutrients, we predict that ApNEAAT1 will localize to the maternal follicular epithelium (the membrane surrounding each embryo) throughout embryogenesis.

## Results

### ApNEAAT1 protein does not colocalize with Buchnera during asexual embryogenesis

**Fig. 2:**
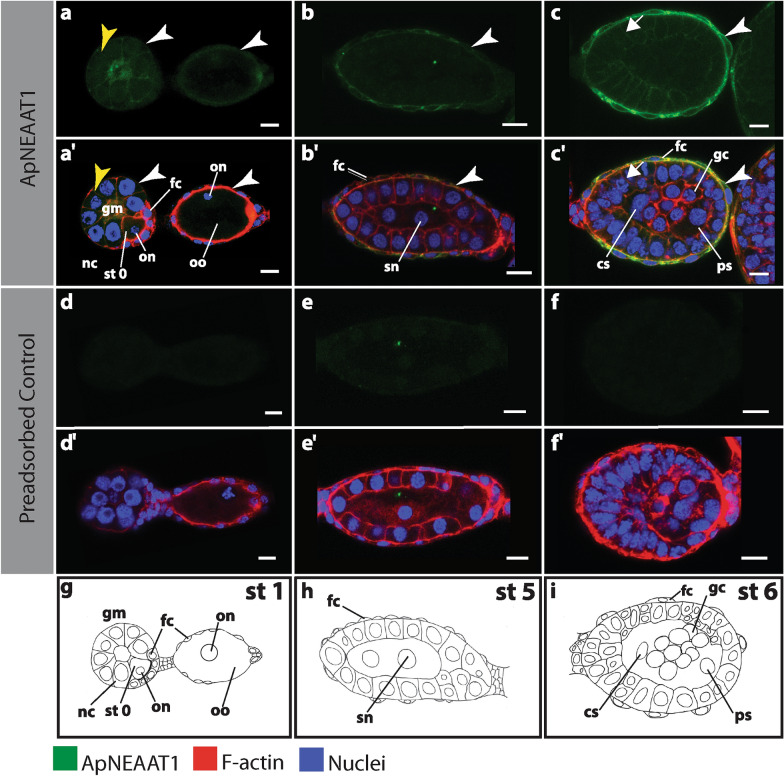
ApNEAAT1 localization in early developing embryos prior to symbiont transmission (germarium, stages 0, 1, 5 and 6). *Buchnera aphidicola* is not present in the germarium, oocytes and developing embryos before stage 6. Signals representing ApNEAAT1 immunoactivity are shown in green, F-actin (Phalloidin) is in red, and nuclei (DAPI) are in blue (color key below figure). Confocal images **a–c** show ApNEAAT1 antibody staining and **a’–c’** show merged results for ApNEAAT1 antibody, F-actin, and nuclei. Confocal images **d–f** are preadsorbed controls showing the antibody signal and **d’–f’** are preadsorbed controls showing the merged results for ApNEAAT1 antibody, F-actin, and nuclei. **g–i** Illustrations of each embryonic stage. White arrowheads mark ApNEAAT1 antibody localization to the maternal follicular epithelium; yellow arrowheads mark localization to germaria membranes; arrows indicate somatic cell membrane localization. Scale bars = 10 µm. *cs* central syncytium, *fc* follicle cells, *gc* germ cells, *gm* germarium, *nc* nurse cells, *on* oocyte nucleus, *oo* oocyte, *ps* posterior syncytium, *sn* syncytial nucleus, *st* stage

**Fig. 3:**
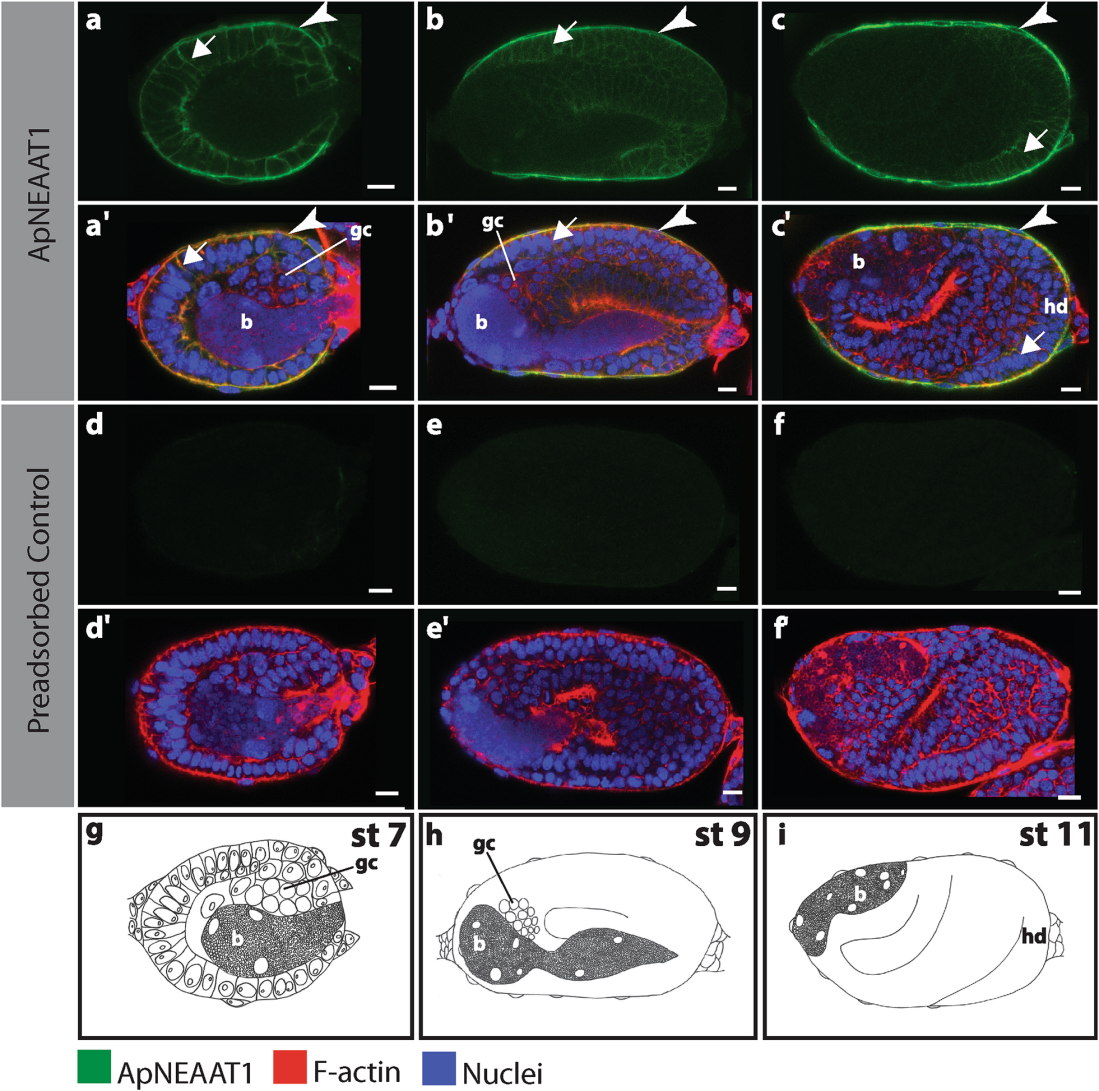
ApNEAAT1 localization in embryos during symbiont transmission (stages 7, 9, and 11). Signals representing ApNEAAT1 immunoactivity are shown in green, F-actin (Phalloidin) is in red, and nuclei (DAPI) are in blue (color key below figure). Confocal images **a–c** show ApNEAAT1 antibody staining and **a’–c’**show merged results for ApNEAAT1 antibody, F-actin, and nuclei. Confocal images **d–f** are preadsorbed controls showing the antibody signal and **d’–f’** are preadsorbed controls showing the merged results for ApNEAAT1 antibody, F-actin, and nuclei. **g–i** Illustrations of each embryonic stage. White arrowheads mark ApNEAAT1 antibody localization to the maternal follicular epithelium; arrows indicate somatic cell membrane localization. Scale bars = 10 µm. *b* endosymbiotic bacteria *Buchnera*, *gc* germ cells, *hd* head, *st* stage

**Fig. 4:**
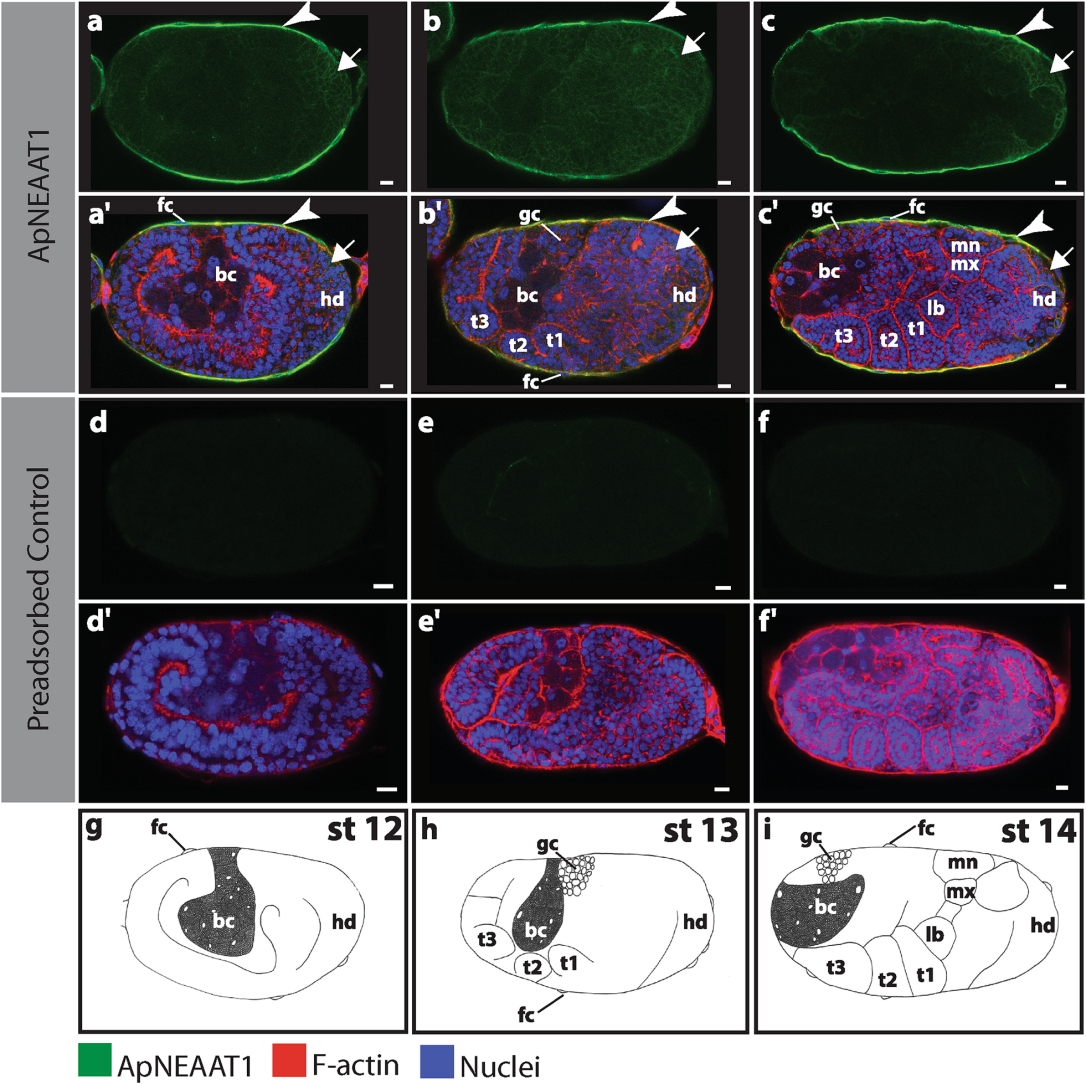
ApNEAAT1 localization in embryos during bacteriocyte cellularization (stages 12, 13, and 14). Signals representing ApNEAAT1 immunoactivity are shown in green, F-actin (Phalloidin) is in red, and nuclei (DAPI) are in blue (color key below figure). Confocal images **a–c** show ApNEAAT1 antibody staining and **a’–c’** show merged results for ApNEAAT1 antibody, F-actin, and nuclei. Confocal images **d–f** are preadsorbed controls showing the antibody signal and **d’–f’** are preadsorbed controls showing the merged results for ApNEAAT1 antibody, F-actin, and nuclei. **g–i** Illustrations of each embryonic stage. White arrowheads mark ApNEAAT1 antibody localization to the maternal follicular epithelium; arrows indicate somatic cell membrane localization. Scale bars = 10 µm. *bc* bacteriocyte, *fc* follicle cells, *gc* germ cells, *hd* head, *lb* labial segment, *mn* mandible segment, *mx* maxilla segment, *st* stage, *t1–t3* the three thoracic segments

**Fig. 5:**
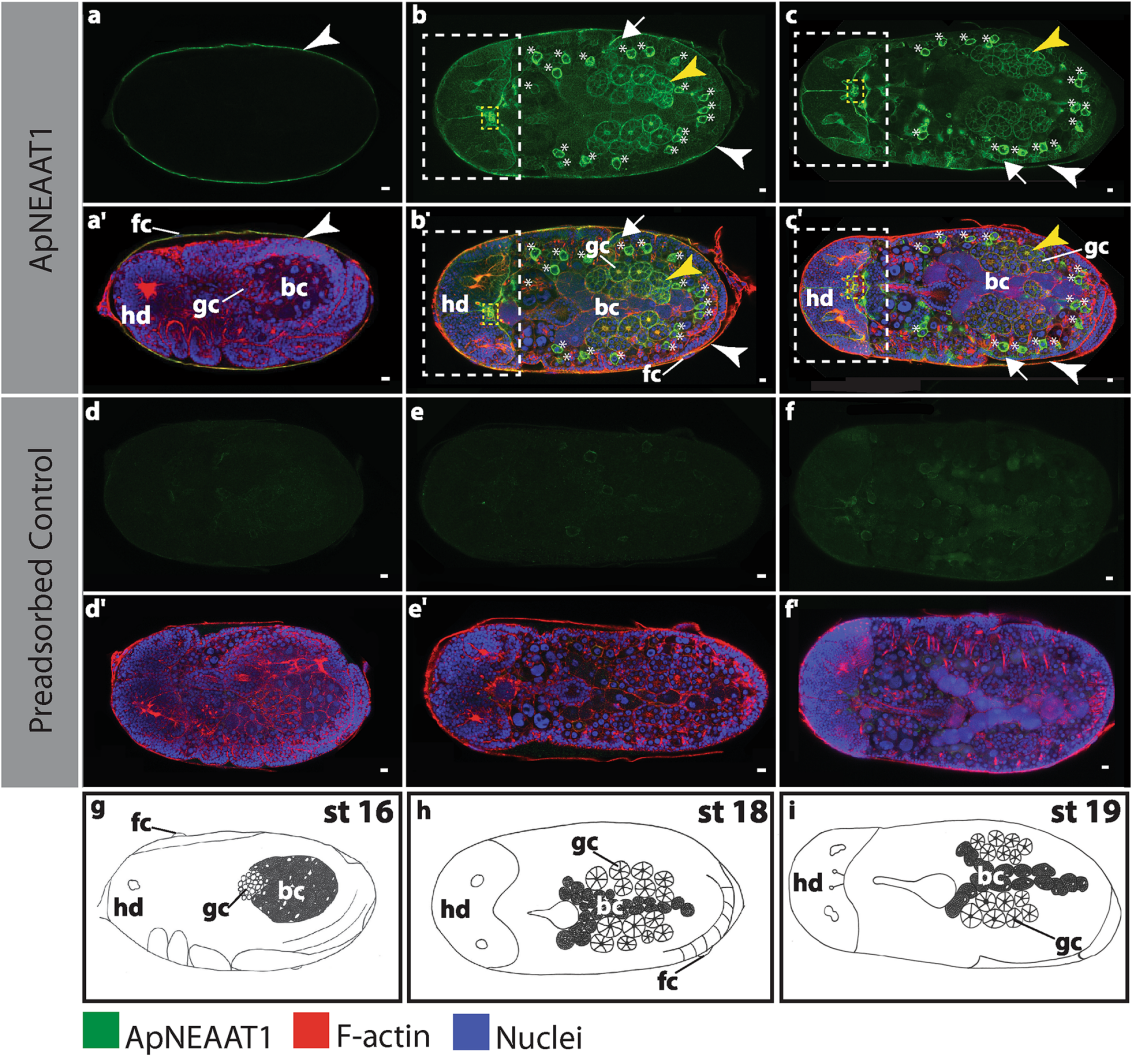
ApNEAAT1 localization in embryos during bacteriome maturation (stages 16, 18, and 19). Signals representing ApNEAAT1 immunoactivity are shown in green, F-actin (Phalloidin) is in red, and nuclei (DAPI) are in blue (color key below figure). Confocal images **a–c** show ApNEAAT1 antibody staining and **a’–c’** show merged results for ApNEAAT1 antibody, F-actin, and nuclei. A magnified view of the head region of panels **b** and **b’** is shown in Additional file 5: Figure S5. Confocal images **d–f** are preadsorbed controls showing the antibody signal and **d’–f’** are preadsorbed controls showing the merged results for ApNEAAT1 antibody, F-actin, and nuclei. **g–i** Illustrations of each embryonic stage. White arrowheads mark ApNEAAT1 antibody localization to the maternal follicular epithelium; yellow arrowheads mark localization to germaria membranes; arrows indicate somatic cell membrane localization; the white dashed rectangle encloses signal appearing in anterior neural structures; the yellow dashed rectangle encloses localization to the corpora cardiaca; asterisks mark localization to prospective hemocytes. Scale bars = 10 µm. *bc* bacteriocyte, *fc* follicle cells, *gc* germ cells, *hd* head, *st* stage

*Buchnera* transmission occurs at stage 7 of asexual embryogenesis; earlier in development *Buchnera* is not present [23] (see Fig. 2, Additional file 1: Figure S1). Across our six replicate experiments, we analyzed 83 embryos between developmental stages 7–19. The position and cellular localization of *Buchnera* changes across development. We never observed ApNEAAT1 signal in the region of embryos occupied by *Buchnera* (Figs. 3, 4, 5, Additional file 2: Figure S2, Additional file 3: Figure S3, Additional file 4: Figure S4). ApNEAAT1 did not localize to *Buchnera*, nor did it localize to the aphid-derived symbiosomal membrane that surrounds individual *Buchnera* cells.

### ApNEAAT1 protein localized to the plasma membrane of the maternal follicular epithelium

**Fig. 6:**
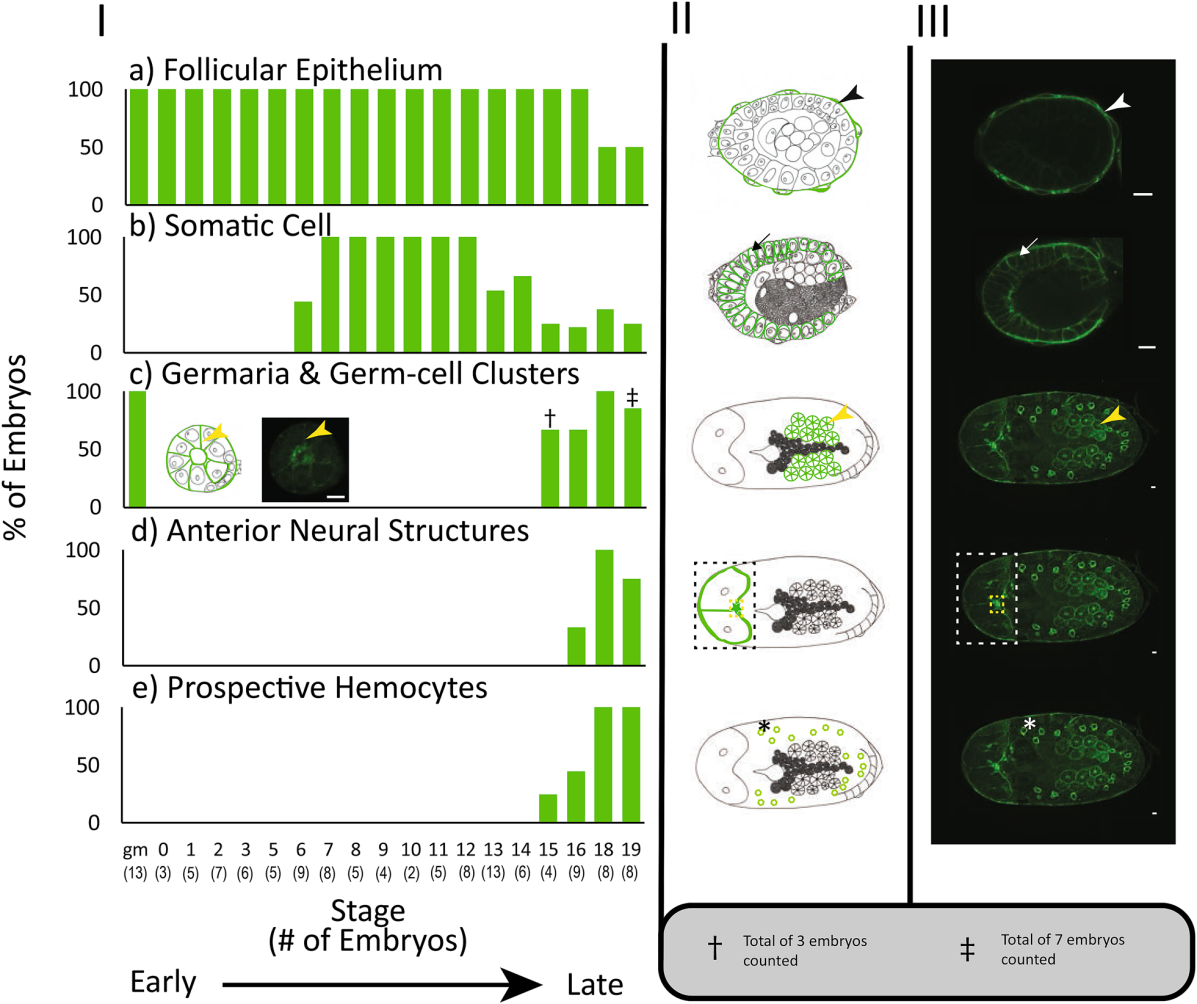
ApNEAAT1 localization patterns across embryonic development. The histograms in column **I** show the percent localization for the five signal patterns (**a–e**) in embryonic stages from germarium (gm) to stage 19. Stages with fewer than 2 embryos captured were omitted. The number of embryos counted for each stage is listed along the x-axis in parentheses, below the indicated stage. Column **II** contains illustrations of each signal pattern, where green represents the ApNEAAT1 signal pattern observed. Column **III** is the confocal image showing the signal pattern (scale bars = 10 μm). White and black arrowheads mark ApNEAAT1 antibody localization to the maternal follicular epithelium; arrows mark somatic cell membrane localization; yellow arrowheads mark localization to germaria membranes; the white and black dashed rectangles enclose signal appearing in anterior neural structures; the yellow dashed rectangles enclose localization to the corpora cardiaca; asterisks mark localization to prospective hemocytes. As a result of embryo orientation or image focal plane, germ-cell clusters/germaria were not visible in all embryos at stages 15 and 19. We report ApNEAAT1 signal only from embryos with visible germ-cell clusters/germaria, and thus, embryo counts were adjusted as indicated by the cross and double-cross symbols (see legend at bottom right of figure)

The maternal follicular epithelium is a single layer of follicle cells that envelop each oocyte or embryo [24, 34]. ApNEAAT1 localized to the inner and outer plasma membrane of follicle cells in all embryos during early (germarium–stage 6; Fig. 2, Additional file 1: Figure S1, white arrowheads) and mid-embryogenesis (stage 7–stage 16; Figs. 3, 4, 5a, aʹ, Additional file 2: Figure S2, Additional file 3: Figure S3, Additional file 4: Figure S4a, aʹ, white arrowheads). In late embryogenesis, stages 18–19, localization of ApNEAAT1 to the plasma membranes of follicle cells was notably less prevalent (Fig. 6a), such that we only observed ApNEAAT1 signal coincident with the plasma membranes of follicle cells in ~ 50% of late-stage embryos (Figs. 5b, bʹ, c, cʹ, Additional file 4: Figure S4b, bʹ, c, cʹ, white arrowheads).

### ApNEAAT1 protein localized to embryonic somatic cell membranes during mid-embryogenesis

The term embryonic somatic cell refers to any cell not associated with the germline [35]. During mid-embryogenesis (stage 6 to stage 16), we observed ApNEAAT1 localization coincident with the plasma membranes of embryonic somatic cells (Figs. 2c, cʹ, 3, 4, 6b, Additional file 1: Figure S1c, cʹ, Additional file 2: Figure S2, Additional file 3: Figure S3, arrows). In approximately 40% of stage 6 embryos, ApNEAAT1 localized to the plasma membranes of somatic cells (Figs. 2c, cʹ, 6b, Additional file 1: Figure S1c, cʹ, arrows). All embryos stages 7–12 had ApNEAAT1-positive somatic cells; however, only about 50% of these embryos exhibited strong ApNEAAT1 signal coincident with somatic cells; the other 50% exhibited weak signal, or strong signal in only a handful of somatic cells located at the posterior end of the embryo. Approximately 40% of embryos stages 13–16 showed strong somatic cell localization. In late-stage embryos (stage 18 to stage 19), somatic cell localization was weakly present in about 30% of the embryos analyzed (Figs. 5, 6b, Additional file 4: Figure S4, arrows). An embryo displaying strong somatic cell membrane signal is shown in Fig. 3, panels a and aʹ (arrows).

### ApNEAAT1 protein localized to the membranes of germaria and embryonic germ-cell clusters

Recall that two types of germaria are represented in our data; those directly dissected from maternal aphids (F1) and those within late-stage embryos (F2) (see Fig. 1). We detected ApNEAAT1 signal localized to embryonic germ-cell clusters in approximately 50% of stage 15 and 16 embryos (Fig. 6c). Strong ApNEAAT1 signals were detected in the germaria within stage 18 and stage 19 embryos (F2 generation; Figs. 5b, bʹ, c, cʹ, Additional file 4: Figure S4b, bʹ, c, cʹ, yellow arrowheads), whereas weak ApNEAAT1 signals were detected in germaria dissected directly from adults (F1 generation; Fig. 2a, aʹ, Additional file 1: Figure S1a, aʹ, yellow arrowheads).

### ApNEAAT1 protein localized to anterior neural structures in late-stage embryos

Two important insect anterior neural structures include the brain and the corpora cardiaca. The corpora cardiaca is a paired neurohemal organ found dorsal to the brain, just below the midpoint, connected to the protocerebrum by axons [36–39]. We observed strong ApNEAAT1 localization just below the midpoint of the brain in ~ 33% of stage 16 embryos and in ~ 88% of stage 18 and 19 embryos (Figs. 5b, bʹ, c, cʹ, 6d, Additional file 4: Figure S4b, bʹ, c, cʹ, Additional file 5: Figure S5, yellow dashed rectangle). We predict this region to be the corpora cardiaca based on its location [38, 40].

The invertebrate blood–brain barrier (BBB) is a selective barrier surrounding the brain [41, 42]. We found that ApNEAAT1 localized to a membrane surrounding the brain in ~ 33% of stage 16 embryos and in ~ 88% of stage 18 and 19 embryos (Figs. 5b, bʹ, c, cʹ, 6d, Additional file 4: Figure S4b, bʹ, c, cʹ, white dashed rectangle).

### ApNEAAT1 protein localized to hemocytes in late-stage embryos

Hemocytes are a type of insect cell involved in the innate immune response, protecting the insect from potentially harmful microbes [43–45]. In stages 18 and 19, as well as a small portion of stage 15 (25%) and 16 (44%) embryos, we observed ApNEAAT1 localization to a polygonal-shaped cell approximately 15–20 μm in diameter located near the periphery of the embryo that we predict to be a type of hemocyte (Figs. 5b, bʹ, c, cʹ, 6e, Additional file 4: Figure S4b, bʹ, c, cʹ, asterisks). To test our hypothesis that these polygonal-shaped cells are hemocytes, we extracted and immunostained hemolymph from late-stage embryos. Our analysis revealed polygonal-shaped cells approximately 15–20 μm in diameter that exhibited the same ApNEAAT1 localization pattern as the prospective hemocyte cells we observed within stage 18 and 19 embryos (see Additional file 6: Figure S6).

### Replication and experimental controls

The patterns described were repeatable across all six replicate experiments. For assessment of immunocytochemistry specificity, a negative control (containing only the secondary antibody) and a peptide-preadsorbed control were utilized to detect non-specific signals. All negative control samples were signal free (Additional file 7: Figure S7a, aʹ). For the preadsorbed control, ApNEAAT1 signals were not detected in the majority of embryo samples. However, we did observe weak background signal in almost all preadsorbed control embryo samples after stage 15 (see Fig. 5e, f). As validation of the experimental procedure and sufficiency for antibody penetration into the embryos, we used ApVas as a positive control (see Additional file 7: Figure S7b, bʹ). ApVas is a well-characterized marker specific to *A. pisum* primordial germ cells [46]. Any stages where less than two embryos were captured were excluded from analysis (stage 4 and 17).

To increase the accessibility of our data for red-green color-blind readers, we include a set of supplemental figures (Additional file 1: Figures S1, Additional file 2: Figure S2, Additional file 3: Figure S3, Additional file 4: Figure S4). These figures compliment main Figs. 2, 3, 4, 5, showing the same embryos and ApNEAAT1 signal patterns.

## Discussion

### Protein localization patterns differ between maternal and embryonic bacteriomes

Previously, the non-essential amino acid transporter ApNEAAT1 was shown to localize to the symbiosomal membrane and plasma membrane of bacteriocytes isolated from adult asexual *A. pisum* [30]. In contrast, we observed that ApNEAAT1 is absent from the symbiosomal and bacteriocyte membranes of asexual *A. pisum* embryos (Figs. 3, 4, 5, Additional file 2: Figure S2, Additional file 3: Figure S3, Additional file 4: Figure S4). Our finding that ApNEAAT1 localization differs between maternal and embryonic bacteriomes provides additional evidence that the bacteriome is not equivalent across aphid development. This finding is consistent with a collection of earlier studies that found differences in protein localization [2, 21], gene expression [2], and expression of *Buchnera* proteins and small RNAs [20]. Of particular interest are the differences between maternal and embryonic expression that we reported previously for the glutamine transporter ApGLNT1 [2]. ApGLNT1 localizes in the maternal bacteriome to the plasma membrane of bacteriocyte cells where we proposed that it functions to regulate amino acid biosynthesis by substrate feedback inhibition [47]. In contrast, in the embryonic bacteriome ApGLNT1 does not localize to the membrane of bacteriocytes [2]. It is now clear that the *A. pisum* embryonic and maternal bacteriomes are not equivalent. However, the developmental point at which the embryonic bacteriome reaches maturity remains unknown.

The finding that both ApGLNT1 [2, 47] and ApNEAAT1 [30, and this study] do not show the same patterns of localization in maternal and embryonic bacteriomes suggests that other amino acid transporters must function at the symbiotic interface in the *A. pisum* embryonic bacteriome. Expansion of amino acid transporter families has been reported for several insect lineages that host bacteriome-associated endosymbionts, revealing the importance of these transporters to the symbiosis [48, 49]. Sternorrhynchan insects, feeding on phloem sap, were found to have large expansions of amino acid/auxin permease transporter (AAAP, transporter classification (TC) #2.A.18) and amino acid polyamine organocation transporter (APC, TC #2.A.3) families [48]. Host insect genome-encoded amino acid transporters are crucial for facilitating nutritional endosymbioses between sternorrhynchan insects and their symbionts because most bacterial symbionts of sternorrhynchans have few to no amino acid transporters encoded in their genomes [18, 50–55]. Notably, *A. pisum* is the only sternorrhynchan for which amino acid transporters belonging to these expanded families have been functionally characterized [30, 47]. Price et al*.* [22, 47] identified 40 putative amino acid transporters (belonging to the APC superfamily) in the *A. pisum* genome, five of which were found to be highly expressed in the adult asexual *A. pisum* bacteriome. Based on the work we present here and our earlier immunolocalization of ApGLNT1 during embryogenesis, we speculate that a different set of *A. pisum’s* 40 APC family transporters is highly expressed in the embryonic bacteriome.

### ApNEAAT1 provisions small non-essential amino acids to developing oocytes and embryos

Nutrient provisioning has been reasoned to occur from the maternal hemolymph across the maternal follicular epithelium of embryos [2, 24, 26, 56]. We observed localization of ApNEAAT1 to the maternal follicular epithelium (Figs. 2, 3, 4, 5, 6a, Additional file 1: Figure S1, Additional file 2: Figure S2, Additional file 3: Figure S3, Additional file 44: Figure S4, white arrowheads) and therefore predict that ApNEAAT1 transports small non-essential amino acids from maternal hemolymph to supply developing embryos throughout development. The *A. pisum* glutamine transporter, ApGLNT1, was previously found to exhibit a similar signal pattern, also localizing to the maternal follicular epithelium [2]. However, with regard to nutrient provisioning, ApNEAAT1 differs from ApGLNT1 in two ways. First, ApNEAAT1 localizes to germaria and germ-cell clusters, and therefore additionally appears to provision small non-essential amino acids to the developing germline (Figs. 2, 5, 6c, Additional file 1: Figure S1, Additional file 4: Figure S4, yellow arrowheads). Second, ApNEAAT1 localizes to embryonic somatic cell membranes (Figs. 2–5, Additional file 1: Figure S1, Additional file 2: Figure S2, Additional file 3: Figure S3, Additional file 4: Figure S4, arrows) suggesting that it is required to exchange amino acids between embryonic cells. ApNEAAT1 provisioning of non-essential amino acids may be especially important for embryos containing *Buchnera*, since *Buchnera* cannot synthesize three non-essential amino acids that ApNEAAT1 transports and therefore requires host supply of proline, serine, and alanine [18, 30].

### ApNEAAT1 provisions anterior neural structures in late-stage embryos with small non-essential amino acids

Two important insect anterior neuroendocrine structures include the brain and the corpora cardiaca. The neuroendocrine system controls hormone production and secretion. The corpora cardiaca is involved in the release of hormones synthesized by neurosecretory cells [36–39]. Specifically, the corpora cardiaca synthesizes and stores adipokinetic hormones, which are involved in regulating energy homeostasis [57, 58]. Based on the neural anatomy of the green peach aphid *Myzus persicae*, and fruit fly *Drosophila melanogaster*, the corpora cardiaca is the structure that most closely resembles the ApNEAAT1-positive signal observed below the midpoint of the brain in late-stage *A. pisum* embryos [37, 38, 40] (Figs. 5b, bʹ, c, cʹ, 6d, Additional file 4: Figure S4b, bʹ, c, cʹ, Additional file 5: Figure S5, yellow dashed rectangle). We predict that ApNEAAT1 provisions the corpora cardiaca with small non-essential amino acids and may additionally perform roles pertaining to energy homeostasis.

ApNEAAT1 is a solute carrier transporter related to the mammalian solute carrier 36 (SLC36) family [22, 59]. Solute carrier transporters are known to be responsible for the transport of amino acids in and out of the brain via the blood–brain barrier (BBB) [41]. Additionally, the amino acids transported by ApNEAAT1 play important roles in brain function, such as acting as inhibitory neurotransmitters and/or maintaining neuronal homeostasis [60–66]. Therefore, we predict that the localization of ApNEAAT1 to a membrane surrounding the brain corresponds to the BBB membrane (Figs. 5b, c, 6d, Additional file 4: Figure S4b, c, white dashed rectangle). The BBB of insects differs from that of vertebrates [41, 42, 67]. Rather than separating the brain from blood, the insect BBB must surround the brain to form a separation from the hemolymph, which is free flowing within the body cavity [41, 42, 67]. We predict that ApNEAAT1 provisions small non-essential amino acids to the brain and related anterior neural structures across the BBB. ApNEAAT1, a bidirectional transporter driven by amino acid concentration gradients, may also help maintain homeostasis of amino acid concentrations in the brain.

### ApNEAAT1 performs roles in immunity

The innate immune response defends against microbial pathogens [45, 68]. Cellular defense, one type of innate immune response, is mediated by “blood” cells that destroy potentially harmful microbes via phagocytosis (engulfment of particles); in invertebrates these cells are called hemocytes [43–45]. In late-stage embryos, we found that ApNEAAT1 localized to cells that were polygonal in shape and approximately 15–20 μm in diameter (Figs. 5b, bʹ, c, cʹ, 6e, Additional file 4: Figure S4b, bʹ, c, cʹ, asterisks). Based on their morphology we reasoned that these cells belong to one of three possible cell types: tracheal cells [69–71], oenocytes [40], or hemocytes [43, 44, 72–74]. Tracheal cells are part of the tracheal system, which acts to supply oxygen to various target organs; tracheal cells specifically sense and respond to the oxygen conditions of target organs [69]. Oenocytes are involved in lipid processing and detoxification [75]. Both tracheal cells and oenocytes are typically anchored to internal structures [40, 69, 70, 75]. In contrast, hemocytes circulate in the hemolymph [44, 76, 77]. Thus, we extracted and immunostained hemolymph from late-stage embryos to identify if cells present in the hemolymph stain positively for the ApNEAAT1 antibody. Six cell types have been previously identified from *A. pisum* hemolymph: four distinct hemocytes and two additional cell categories, spherulocytes and wax cells, which are also involved in the aphid immune response [43, 44]. The non-hemocyte cells known to be present in *A. pisum* hemolymph can be ruled out as candidates for the ApNEAAT1-positive cell type based on their localization within aphid bodies: spherulocytes are found just under the cuticle, not deep into the body, while wax cells localize to the base of the cornicles (tube-like projections at the posterior end of the aphid abdomen) [44]. Our immunostained hemolymph revealed ApNEAAT1-positive cells with signal patterns and morphology that matched the polygonal-shaped cells we observed in late-stage whole embryos (see Additional file 6: Figure S6). On the basis of ApNEAAT1 localization to hemocytes, we propose that ApNEAAT1 may perform a role in aphid innate immunity.

## Conclusions

ApNEAAT1 does not localize to the symbiosomal or bacteriocyte membranes of asexual *A. pisum* embryos. This difference in ApNEAAT1 localization between the embryonic and maternal bacteriomes is consistent with earlier work, providing further evidence that the embryonic and adult bacteriomes are distinct [2, 20, 21, 78]. During embryogenesis, instead of functioning at the *A. pisum*/*Buchnera* interface, ApNEAAT1 appears to perform a diverse set of roles that include nutrient provisioning to embryos across the maternal follicular epithelium, nutrient provisioning to the germline and anterior neural structures, and innate immune defense.

## Methods

### Aphids

*A. pisum* cultures of line LSR1 were raised on *Vicia faba* and incubated at 20 °C under a 16-h light/8-h dark cycle. Ovaries of wingless, asexual adult female aphids were dissected for oocyte and embryo collection. Dissections were performed in phosphate-buffered saline (PBS; 10 mM phosphate buffer, 154 mM NaCl, pH 7.4; Sigma). Embryos were staged according to Miura et al*.* [23].

### Preparation of Anti-ApNEAAT1 antibody

We used the anti-ApNEAAT1 antibody purified from rabbit sera as described by Feng et al*.* [30]. This antibody was raised against a synthetic peptide corresponding to amino acids 4–20 of ApNEAAT1 plus a C-terminal cysteine (SLSLTGIGPPSDTKDQK-C).

### Fluorescent immunostaining in oocytes and embryos

Dissected ovaries composed of developing oocytes and embryos were fixed in 3.65% formaldehyde in PBS for 20 min. Immunostaining followed the protocol of Chang et al*.* [25] but omitted the H_2_O_2_ treatment, and, to increase antibody penetration, late-stage embryos (stages 17–19) following fixation were incubated in Proteinase K (1 μg/ml) for 10 min at room temperature [79]. Oocytes and embryos were incubated overnight at 4 °C in the rabbit anti-ApNEAAT1 monospecific antibody at a 1:20 dilution. Next, to fluorescently label the rabbit anti-ApNEAAT1 IgGs, samples were incubated at 4 °C overnight in Alexa Fluor 633-conjugated goat anti-rabbit IgG antibody (Invitrogen) at a dilution of 1:500. A negative control containing only the secondary antibody and a preadsorbed control were utilized to detect non-specific signals. An ApVas1 (ApVas)-positive control at 1:200 dilution was used to confirm the effectiveness of the protocol [25, 46]. We counterstained nuclei and F-actin, with 4′,6-diamidino-2-phenylindole (DAPI) and phalloidin-tetramethylrhodamine B isothiocyanate (phalloidin-TRITC), respectively, for 1 h at room temperature. Prior to mounting, samples were incubated at 4 °C overnight in 70% glycerol diluted with PBS. A Leica TCS SP5 laser scanning confocal microscope in the University of Miami, Department of Biology Microscopy Core Facility was used to acquire images. Images of germaria through developmental stage 19 were collected for analysis. Control treatments were run in parallel. The experiment was performed six times.

### Fluorescent immunostaining of embryonic hemolymph

Late-stage embryos (stage 18 and 19) were collected from adult asexual *A. pisum* (LSR1) in ice-cold PBS (10 mM phosphate buffer, 154 mM NaCl, and pH 7.4). Embryos were transferred to poly-L-lysine-coated glass microscope slides, punctured in the thorax using a needle, and then gently squeezed with forceps to extract hemolymph. The embryo husks were then removed from the glass slides, and the extracted hemolymph was allowed to fully dry. The adhered cells were next fixed in 3.65% formaldehyde in PBS for five minutes. Slides were then washed with 0.2% PTX [PBS with Triton X-100 (Alfa Aesar)] three times for five minutes each. Cells were blocked in 1 × Blocking Reagent (Roche) overnight at 4 °C and then incubated overnight at 4 °C in the rabbit anti-ApNEAAT1 monospecific antibody at a 1:500 dilution. Slides were washed with 0.2% PTX four times for five minutes each. Cells were blocked again in 1 × Blocking Reagent for 30 min at room temperature. Samples were then incubated for one hour at room temperature in Alexa Fluor 633-conjugated goat anti-rabbit IgG antibody (Invitrogen) at a dilution of 1:500. The secondary antibody was washed off using 0.2% PTX (three washes, 5 min each). We counterstained nuclei and F-actin, with DAPI and phalloidin-TRITC, respectively, for 30 min at room temperature. The counterstains were washed off using 0.2% PTX (four washes, five minutes each). One drop of 70% glycerol/PBS was applied to the microscope slide prior to placing the coverslip over the cells. A negative control containing only the secondary antibody (no primary antibody) and a preadsorbed control were utilized to detect non-specific signals. ApNEAAT1 signals were not detected in the negative or preadsorbed controls. A Leica TCS SP5 laser scanning confocal microscope in the University of Miami, Department of Biology Microscopy Core Facility was used to acquire images.

## Supplementary information

**Additional file 1: Figure S1.** ApNEAAT1 localization in early developing embryos prior to symbiont transmission (germarium, stages 0, 1, 5, and 6). *Buchnera aphidicola* is not present in the germarium, oocyte, and developing embryos before stage 6. Signals representing ApNEAAT1 immunoactivity are shown in red, F-actin (Phalloidin) is in blue, and nuclei (DAPI) are in white (color key below figure). Confocal images (**a-c**) show ApNEAAT1 antibody staining and (**a’–c’**) show merged results for ApNEAAT1 antibody, F-actin, and nuclei. Confocal images (**d–f**) are preadsorbed controls showing the antibody signal and (**d’–f’**) are preadsorbed controls showing the merged results for ApNEAAT1 antibody, F-actin and nuclei. Panels (**g–i**) are illustrations of each embryonic stage. White arrowheads mark ApNEAAT1 antibody localization to the maternal follicular epithelium; yellow arrowheads mark localization to germaria membranes; arrows indicate somatic cell membrane localization. Scale bars =10 µm. *cs* central syncytium, *fc* follicle cells, *gc* germ cells, *gm* germarium, *nc* nurse cells, *on* oocyte nucleus, *oo* oocyte, *ps* posterior syncytium, *sn* syncytial nucleus, *st* stage.

**Additional file 2: Figure S2.** ApNEAAT1 localization in embryos during symbiont transmission (stages 7, 9, and 11). Signals representing ApNEAAT1 immunoactivity are shown in red, F-actin (Phalloidin) is in blue, and nuclei (DAPI) are in white (color key below figure). Confocal images (**a–c**) show ApNEAAT1 antibody staining and (**a’–c’**) show merged results for ApNEAAT1 antibody, F-actin, and nuclei. Confocal images (**d–f**) are preadsorbed controls showing the antibody signal and (**d’–f’**) are preadsorbed controls showing the merged results for ApNEAAT1 antibody, F-actin, and nuclei. Panels (**g-i**) are illustrations of each embryonic stage. White arrowheads mark ApNEAAT1 antibody localization to the maternal follicular epithelium; arrows indicate somatic cell membrane localization. Scale bars = 10 µm. *b* endosymbiotic bacteria *Buchnera*, *gc* germ cells, *hd* head, *st* stage.

**Additional file 3: Figure S3.** ApNEAAT1 localization in embryos during bacteriocyte cellularization (stages 12, 13, and 14). Signals representing ApNEAAT1 immunoactivity are shown in red, F-actin (Phalloidin) is in blue, and nuclei (DAPI) are in white (color key below figure). Confocal images (**a–c**) show ApNEAAT1 antibody staining and (**a’–c’**) show merged results for ApNEAAT1 antibody, F-actin, and nuclei. Confocal images (**d–f**) are preadsorbed controls showing the antibody signal and (**d’–f’**) are preadsorbed controls showing the merged results for ApNEAAT1 antibody, F-actin, and nuclei. Panels (**g–i**) are illustrations of each embryonic stage. White arrowheads mark ApNEAAT1 antibody localization to the maternal follicular epithelium; arrows indicate somatic cell membrane localization. Scale bars = 10 µm. *bc* bacteriocyte, *fc* follicle cells, *gc* germ cells, *hd* head, *lb* labial segment, *mn* mandible segment, *mx* maxilla segment, *st* stage, *t1-t3* the three thoracic segments.

**Additional file 4: Figure S4.** ApNEAAT1 localization in embryos during bacteriome maturation (stages 16, 18, and 19). Signals representing ApNEAAT1 immunoactivity are shown in red, F-actin (Phalloidin) is in blue, and nuclei (DAPI) are in white (color key below figure). Confocal images (**a–c**) show ApNEAAT1 antibody staining and (**a’–c’**) show merged results for ApNEAAT1 antibody, F-actin, and nuclei. A magnified view of the head region of panels **b** and **b’ **is shown in Additional file 5: Figure S5. Confocal images (**d–f**) are preadsorbed controls showing the antibody signal and (**d’–f’**) are preadsorbed controls showing the merged results for ApNEAAT1 antibody, F-actin, and nuclei. Panels (**g–i**) are illustrations of each embryonic stage. White arrowheads mark ApNEAAT1 antibody localization to the maternal follicular epithelium; yellow arrowheads mark localization to germaria membranes; arrows indicate somatic cell membrane localization; the white dashed rectangle encloses signal appearing in anterior neural structures; the yellow dashed rectangle encloses localization to the corpora cardiaca; asterisks mark localization to prospective hemocytes. Scale bars = 10 µm. *bc* bacteriocyte, *fc* follicle cells, *gc* germ cells, *hd* head, *st* stage.

**Additional file 5: Figure S5.** ApNEAAT1 localization to anterior neural structures. Panels **a** and **a’ **show a magnified confocal image of a stage 18 embryo head (see Fig. 5b & b**’ **for full embryo image) stained with ApNEAAT1 antibody (green), Phalloidin marking F-actin (red), and DAPI marking nuclei (blue) (see color key below panel **a’**). Panel **a** shows ApNEAAT1 signal only and panel **a’** shows the merged image. The yellow dashed rectangle encloses localization to the corpora cardiaca. Scale bars = 10 µm

**Additional file 6: Figure S6.** ApNEAAT1 localization to hemocytes. Panels **a** and **a’ **show a confocal image of an isolated hemocyte from the hemolymph of a late-stage embryo. Panel** a** shows the ApNEAAT1 antibody channel only and panel **a’ **shows the merged image. The ApNEAAT1 antibody is shown in green, F-actin (Phalloidin) in red, and nuclei (DAPI) in blue (see color key below figure). Panels **b** and **b’ **show a magnified view of a prospective hemocyte (“ApNEAAT1-positive cell”) from within a stage 19 embryo (see Fig. 5c & c’ for full embryo image). A preadsorbed (**c** & **c’**) and negative (**d** & **d’**) control for the hemolymph staining are also shown. Scale bars = 10 µm.

**Additional file 7: Figure S7.** Negative and Positive controls for immunolocalization procedure. Panels **a** and **a’** show confocal images of embryos incubated in only the secondary antibody (negative control). ApVas antibody [46] was used as a positive control to test the effectiveness of the immunostaining protocol used. Panels **b** and **b’** show confocal images of embryos stained with ApVas antibody (positive control). ApNEAAT1 and ApVas antibodies are shown in green, F-actin (Phalloidin) in red, and nuclei (DAPI) in blue (see color key below figure). *gm* germarium, *st* stage. Scale bars = 10 µm.

## Data Availability

Not applicable.

## Abbreviations

AAAP: Amino acid/auxin permease transporters
APC: Amino acid polyamine organocation transporters
ApGLNT1: *Acyrthosiphon pisum* Glutamine transporter 1
ApNEAAT1: *Acyrthosiphon pisum* Non-essential amino acid transporter 1
ApVAS1: *Acyrthosiphon pisum* Vasa 1
BBB: Blood–brain barrier
DAPI: 4′,6-Diamidino-2-phenylindole
IgG: Immunoglobulin G
LSR1: *Acyrthosiphon pisum* Clonal line
PBS: Phosphate-buffered saline
phalloidin-TRITC: Phalloidin-tetramethylrhodamine B isothiocyanate
PTX: Phosphate-buffered saline (PBS) with Triton X-100
SLC36: Solute carrier family 36

## Footnotes

1 *A. pisum* contains telotrophic meroistic ovarioles. The germarium of asexual *A. pisum* comprises two cell types. These include nurse cells and oocytes. Oocytes develop into embryos by omitting the reduction division of meiosis I. In asexual *A. pisum* the transition from oocyte to embryo occurs with the transition from stage 2 to stage 3 of development [23]. The staging of embryogenesis varies across ovarioles; thus, depending on developmental stage, this could represent one oocyte and 10 embryos of increasing developmental age or, it could represent two oocytes, and 9 embryos of increasing developmental age. Thus, we say that each ovariole contains a string of ~ 11 oocytes/embryos.
