# Supplementary figures and images for: Further evidence that mechanisms of host/symbiont integration are dissimilar in the maternal versus embryonic Acyrthosiphon pisum bacteriome
Source: EvoDevo. 2020 Nov 10;11:23. doi: 10.1186/s13227-020-00168-5 (PMC7654044; doi:10.1186/s13227-020-00168-5)

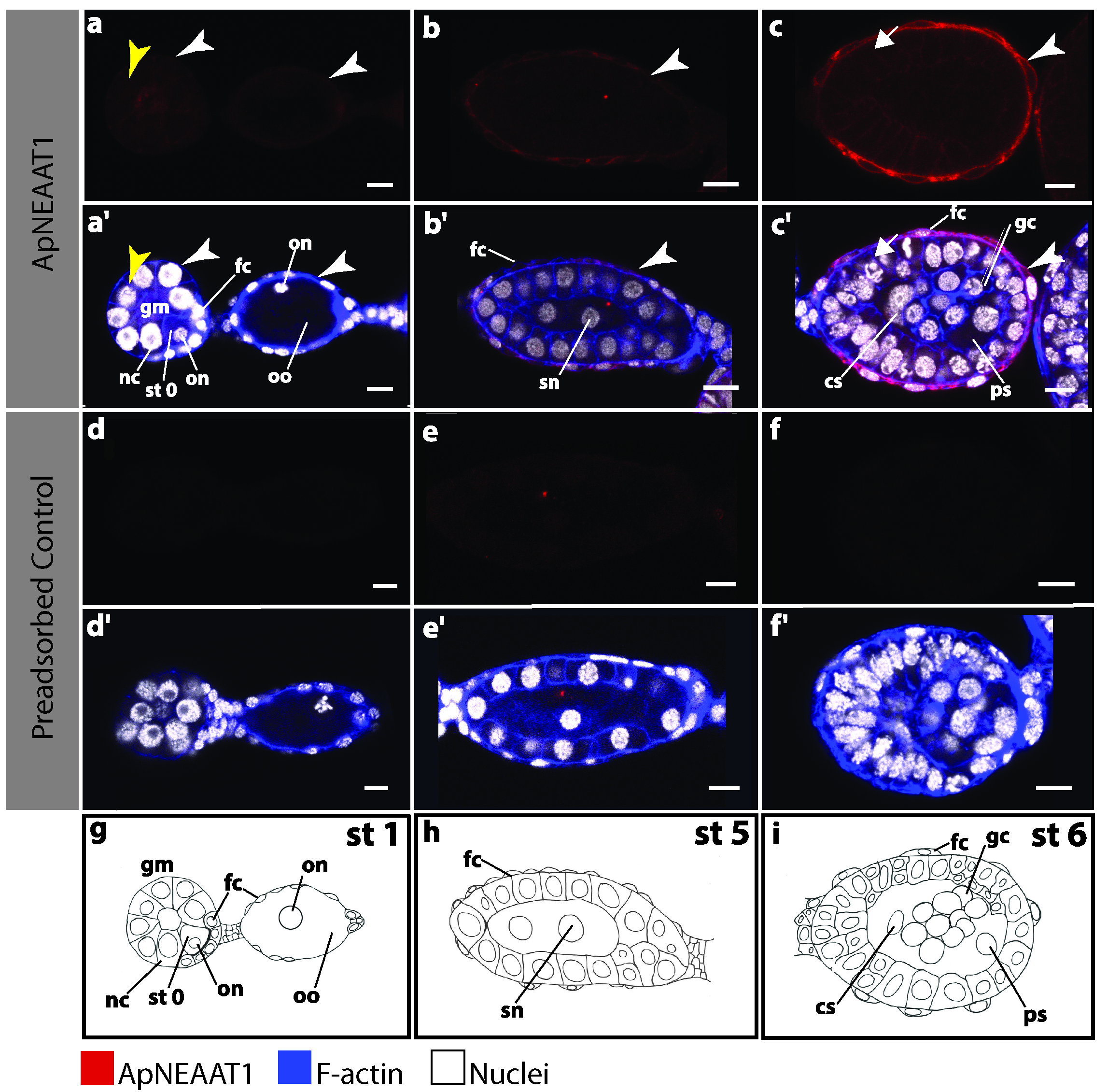

Supplement: Supplementary file 1 — Additional file 1: Figure S1. ApNEAAT1 localization in early developing embryos prior to symbiont transmission (germarium, stages 0, 1, 5, and 6). Buchnera aphidicola is not present in the germarium, oocyte, and developing embryos before stage 6. Signals representing ApNEAAT1 immunoactivity are shown in red, F-actin (Phalloidin) is in blue, and nuclei (DAPI) are in white (color key below figure). Confocal images (a-c) show ApNEAAT1 antibody staining and (a’–c’) show merged results for ApNEAAT1 antibody, F-actin, and nuclei. Confocal images (d–f) are preadsorbed controls showing the antibody signal and (d’–f’) are preadsorbed controls showing the merged results for ApNEAAT1 antibody, F-actin and nuclei. Panels (g–i) are illustrations of each embryonic stage. White arrowheads mark ApNEAAT1 antibody localization to the maternal follicular epithelium; yellow arrowheads mark localization to germaria membranes; arrows indicate somatic cell membrane localization. Scale bars =10 µm. cs central syncytium, fc follicle cells, gc germ cells, gm germarium, nc nurse cells, on oocyte nucleus, oo oocyte, ps posterior syncytium, sn syncytial nucleus, st stage. [file 13227_2020_168_MOESM1_ESM.tif]

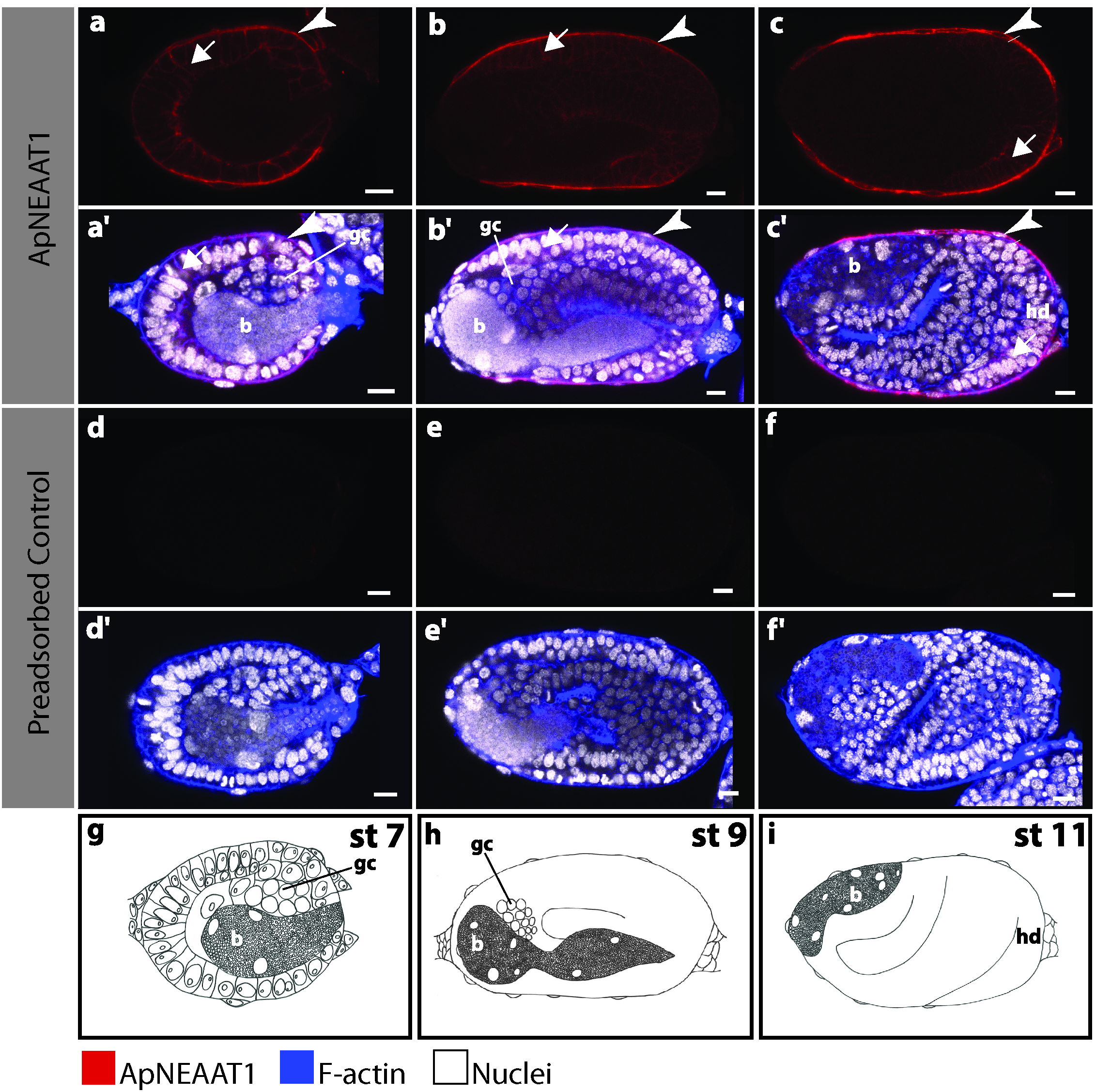

Supplement: Supplementary file 2 — Additional file 2: Figure S2. ApNEAAT1 localization in embryos during symbiont transmission (stages 7, 9, and 11). Signals representing ApNEAAT1 immunoactivity are shown in red, F-actin (Phalloidin) is in blue, and nuclei (DAPI) are in white (color key below figure). Confocal images (a–c) show ApNEAAT1 antibody staining and (a’–c’) show merged results for ApNEAAT1 antibody, F-actin, and nuclei. Confocal images (d–f) are preadsorbed controls showing the antibody signal and (d’–f’) are preadsorbed controls showing the merged results for ApNEAAT1 antibody, F-actin, and nuclei. Panels (g-i) are illustrations of each embryonic stage. White arrowheads mark ApNEAAT1 antibody localization to the maternal follicular epithelium; arrows indicate somatic cell membrane localization. Scale bars = 10 µm. b endosymbiotic bacteria Buchnera, gc germ cells, hd head, st stage. [file 13227_2020_168_MOESM2_ESM.tif]

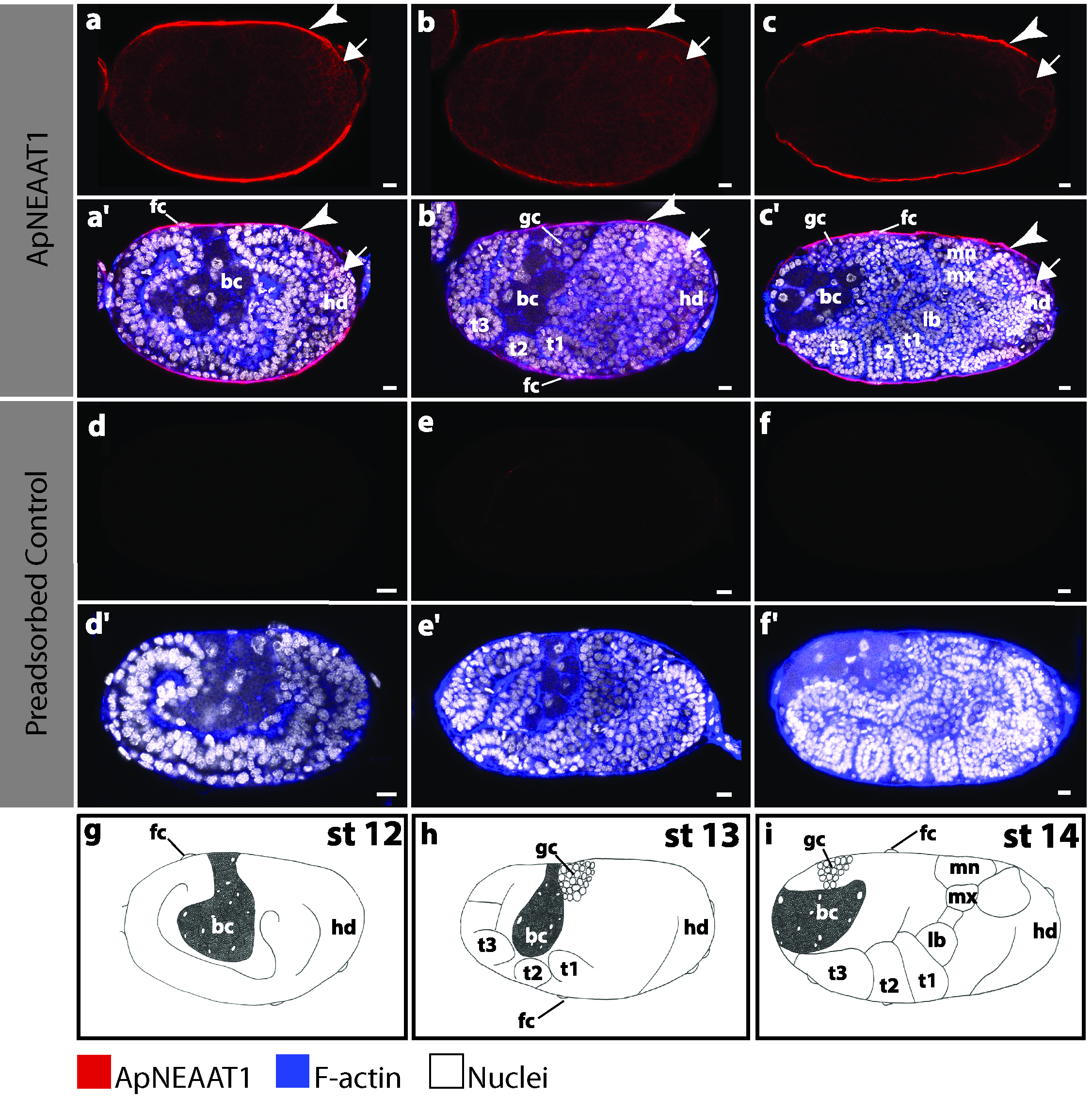

Supplement: Supplementary file 3 — Additional file 3: Figure S3. ApNEAAT1 localization in embryos during bacteriocyte cellularization (stages 12, 13, and 14). Signals representing ApNEAAT1 immunoactivity are shown in red, F-actin (Phalloidin) is in blue, and nuclei (DAPI) are in white (color key below figure). Confocal images (a–c) show ApNEAAT1 antibody staining and (a’–c’) show merged results for ApNEAAT1 antibody, F-actin, and nuclei. Confocal images (d–f) are preadsorbed controls showing the antibody signal and (d’–f’) are preadsorbed controls showing the merged results for ApNEAAT1 antibody, F-actin, and nuclei. Panels (g–i) are illustrations of each embryonic stage. White arrowheads mark ApNEAAT1 antibody localization to the maternal follicular epithelium; arrows indicate somatic cell membrane localization. Scale bars = 10 µm. bc bacteriocyte, fc follicle cells, gc germ cells, hd head, lb labial segment, mn mandible segment, mx maxilla segment, st stage, t1-t3 the three thoracic segments. [file 13227_2020_168_MOESM3_ESM.tif]

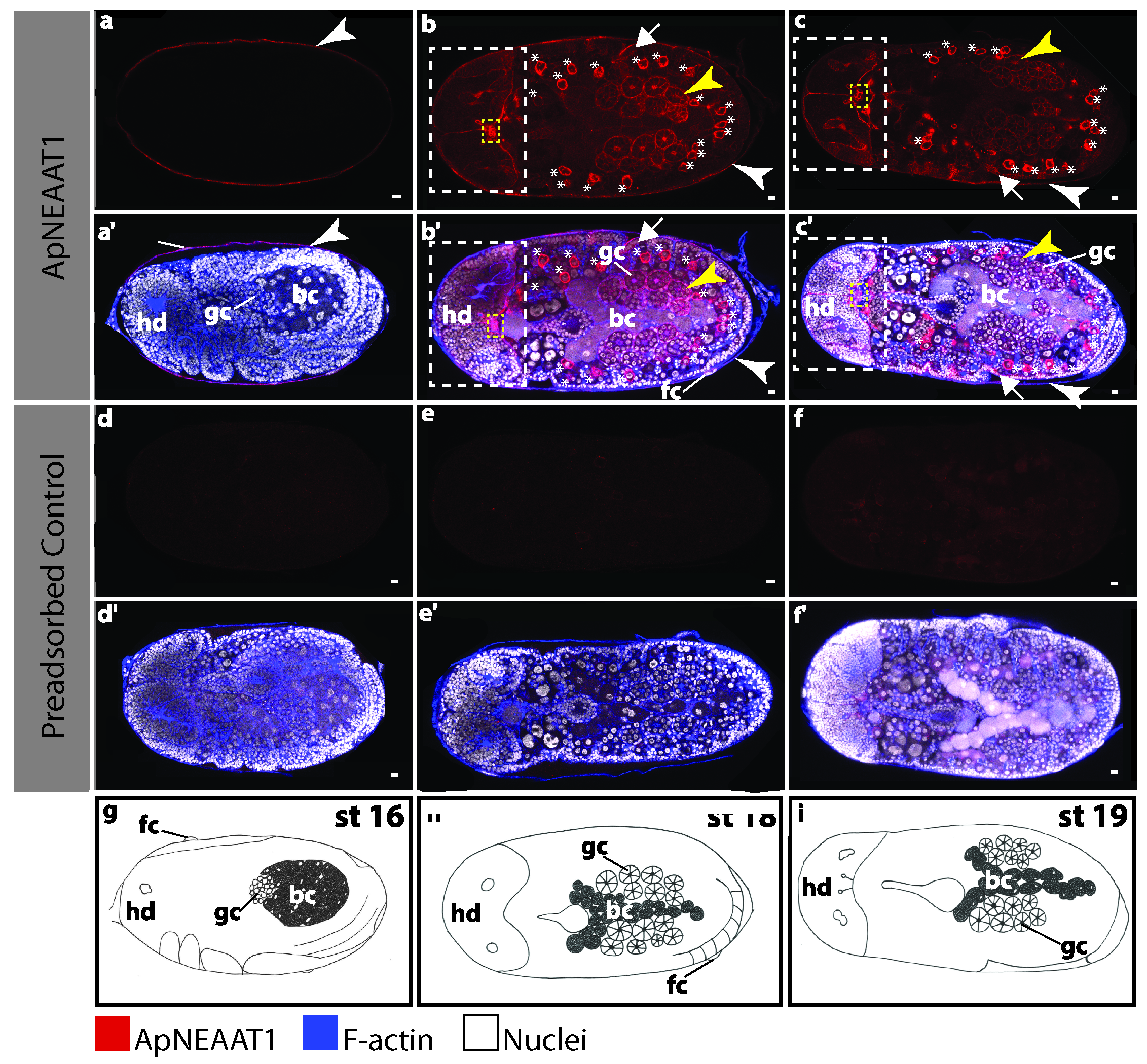

Supplement: Supplementary file 4 — Additional file 4: Figure S4. ApNEAAT1 localization in embryos during bacteriome maturation (stages 16, 18, and 19). Signals representing ApNEAAT1 immunoactivity are shown in red, F-actin (Phalloidin) is in blue, and nuclei (DAPI) are in white (color key below figure). Confocal images (a–c) show ApNEAAT1 antibody staining and (a’–c’) show merged results for ApNEAAT1 antibody, F-actin, and nuclei. A magnified view of the head region of panels b and b’ is shown in Additional file 5: Figure S5. Confocal images (d–f) are preadsorbed controls showing the antibody signal and (d’–f’) are preadsorbed controls showing the merged results for ApNEAAT1 antibody, F-actin, and nuclei. Panels (g–i) are illustrations of each embryonic stage. White arrowheads mark ApNEAAT1 antibody localization to the maternal follicular epithelium; yellow arrowheads mark localization to germaria membranes; arrows indicate somatic cell membrane localization; the white dashed rectangle encloses signal appearing in anterior neural structures; the yellow dashed rectangle encloses localization to the corpora cardiaca; asterisks mark localization to prospective hemocytes. Scale bars = 10 µm. bc bacteriocyte, fc follicle cells, gc germ cells, hd head, st stage. [file 13227_2020_168_MOESM4_ESM.tif]

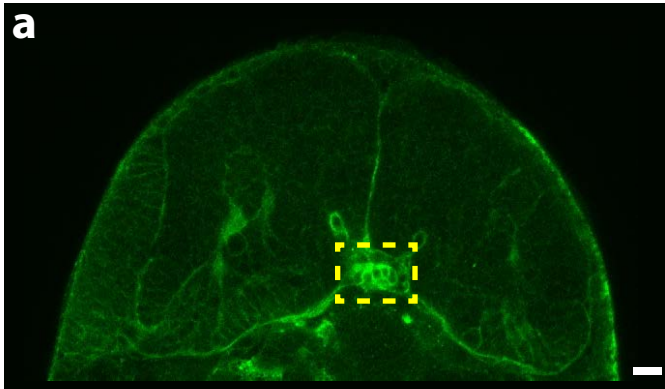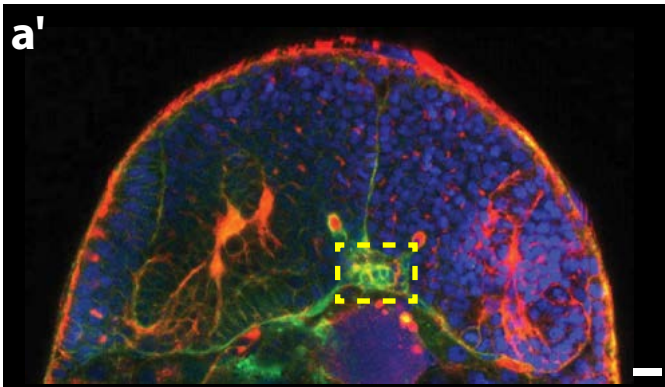

■ ApNEAAT1 ■ F-actin ■ Nuclei

Supplement: Supplementary file 5 — Additional file 5: Figure S5. ApNEAAT1 localization to anterior neural structures. Panels a and a’ show a magnified confocal image of a stage 18 embryo head (see Fig. 5b & b’ for full embryo image) stained with ApNEAAT1 antibody (green), Phalloidin marking F-actin (red), and DAPI marking nuclei (blue) (see color key below panel a’). Panel a shows ApNEAAT1 signal only and panel a’ shows the merged image. The yellow dashed rectangle encloses localization to the corpora cardiaca. Scale bars = 10 µm [file 13227_2020_168_MOESM5_ESM.pdf]

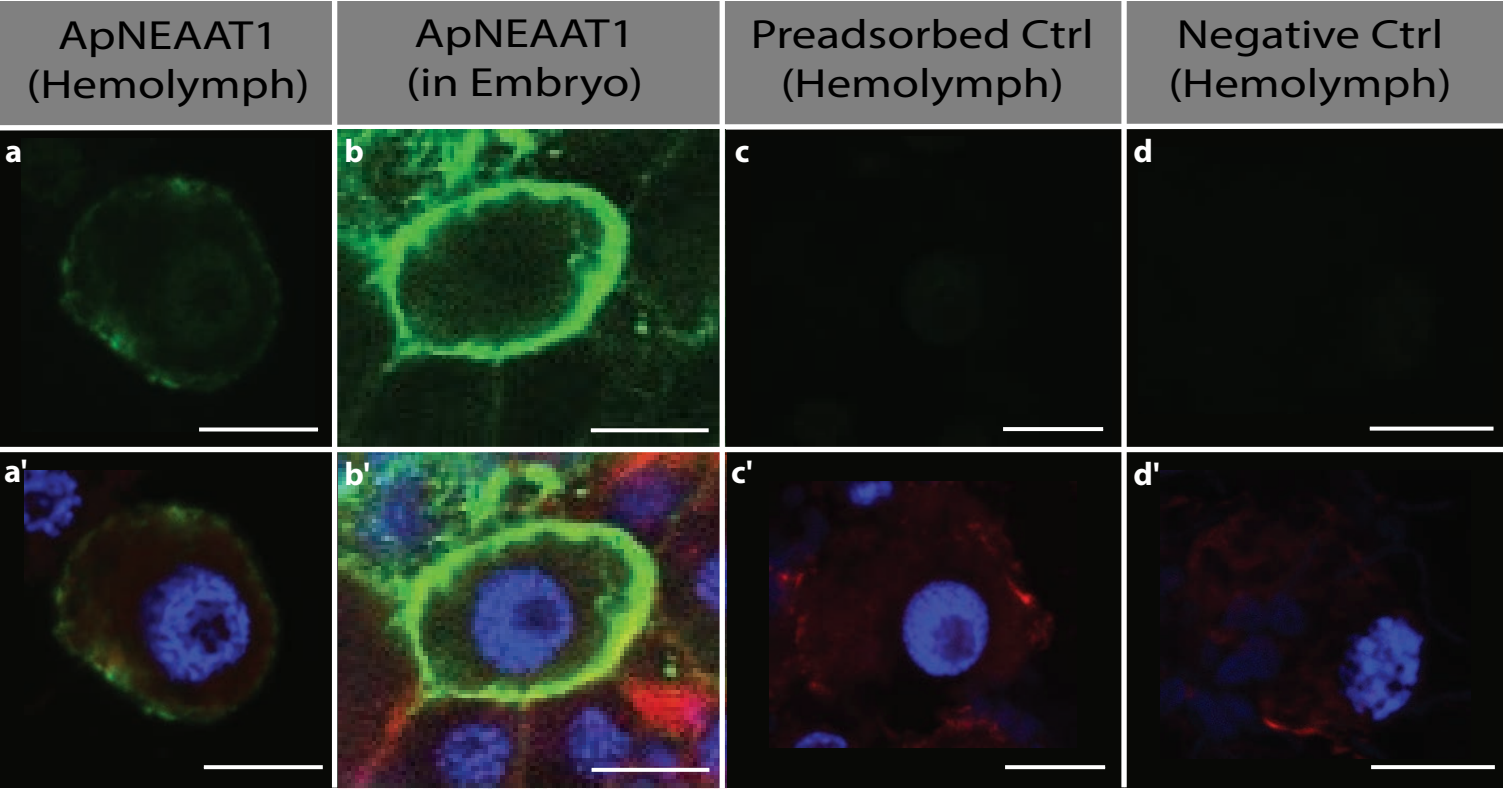

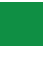 ApNEAAT1   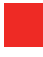 F-actin   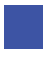 Nuclei

Supplement: Supplementary file 6 — Additional file 6: Figure S6. ApNEAAT1 localization to hemocytes. Panels a and a’ show a confocal image of an isolated hemocyte from the hemolymph of a late-stage embryo. Panel a shows the ApNEAAT1 antibody channel only and panel a’ shows the merged image. The ApNEAAT1 antibody is shown in green, F-actin (Phalloidin) in red, and nuclei (DAPI) in blue (see color key below figure). Panels b and b’ show a magnified view of a prospective hemocyte (“ApNEAAT1-positive cell”) from within a stage 19 embryo (see Fig. 5c & c’ for full embryo image). A preadsorbed (c & c’) and negative (d & d’) control for the hemolymph staining are also shown. Scale bars = 10 µm. [file 13227_2020_168_MOESM6_ESM.pdf]

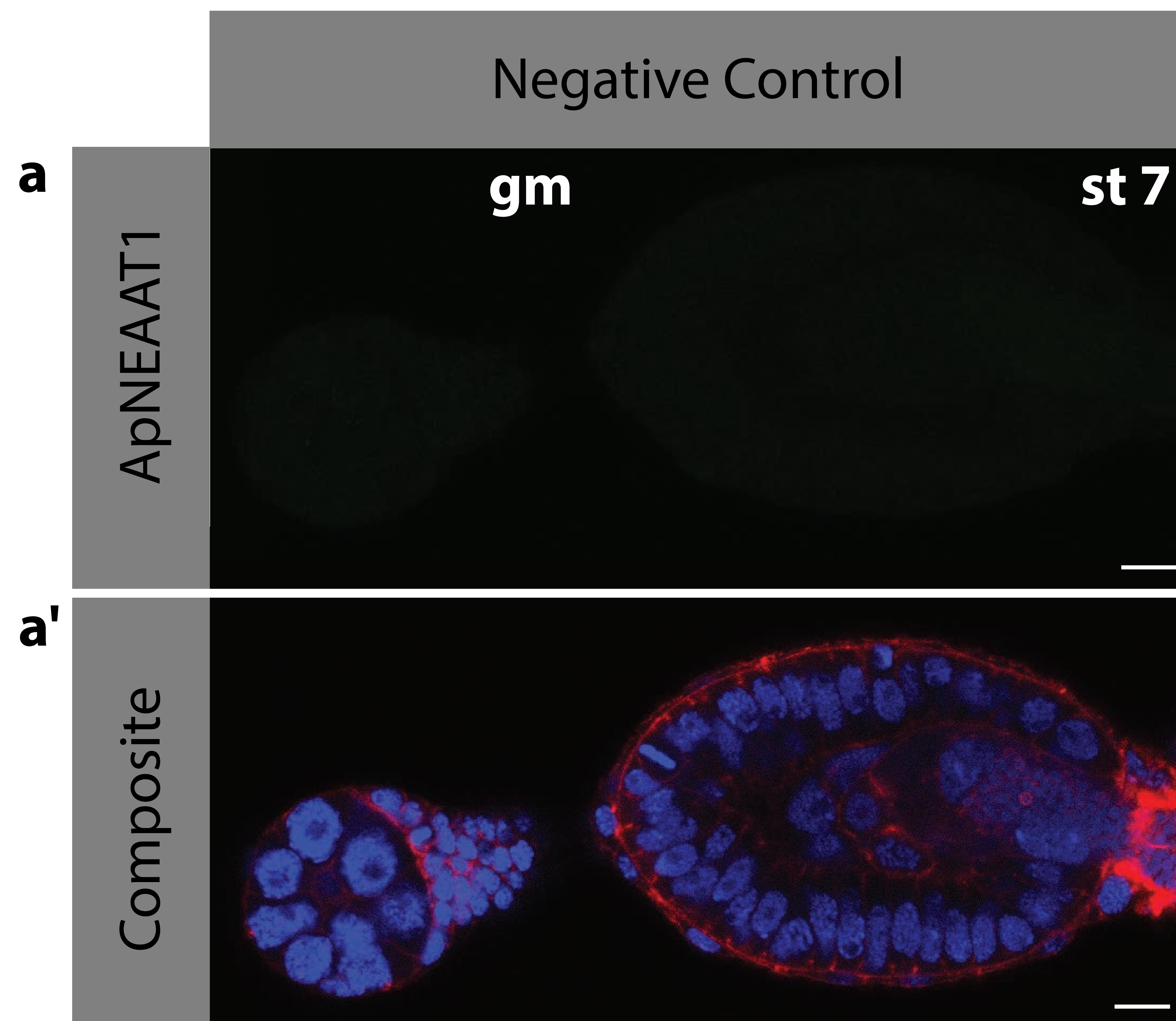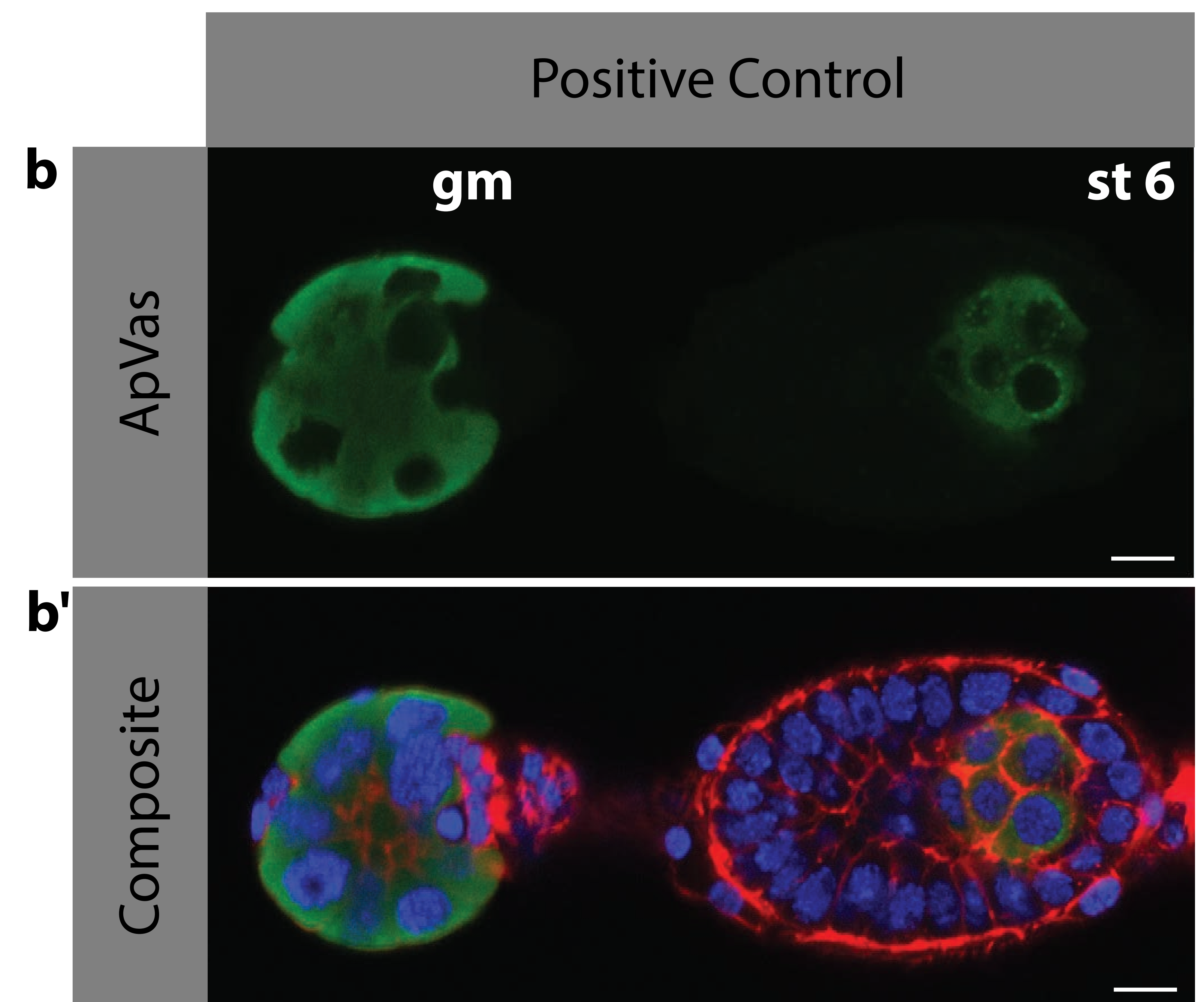

Antibody F-actin Nuclei

Supplement: Supplementary file 7 — Additional file 7: Figure S7. Negative and Positive controls for immunolocalization procedure. Panels a and a’ show confocal images of embryos incubated in only the secondary antibody (negative control). ApVas antibody [46] was used as a positive control to test the effectiveness of the immunostaining protocol used. Panels b and b’ show confocal images of embryos stained with ApVas antibody (positive control). ApNEAAT1 and ApVas antibodies are shown in green, F-actin (Phalloidin) in red, and nuclei (DAPI) in blue (see color key below figure). gm germarium, st stage. Scale bars = 10 µm. [file 13227_2020_168_MOESM7_ESM.pdf]
